# Gambling and governmental responsibilities

**DOI:** 10.1192/bji.2021.8

**Published:** 2021-05

**Authors:** David Skuse

**Affiliations:** Professor of Behavioural and Brain Sciences, Division of Population, Policy and Practice, UCL Great Ormond Street Institute of Child Health, London, UK. Email: d.skuse@ucl.ac.uk

**Keywords:** Gambling, addiction, government, Nigeria, Malaysia

## Abstract

Human beings seem to be genetically predisposed to take risks in the hope of reward, but in gambling the risks often significantly outweigh the rewards. Should societies and governments attempt to regulate gambling, and how? Two papers in this month's issue of *BJPsych International* discuss problem gambling in Nigeria and in Malaysia, and how government and society address it.

Gambling is one of the world's oldest pastimes, with evidence suggesting that it originated sometime during the Old Stone Age, before the days of written history. That fact alone implies that we are genetically predisposed to take risks with the expectation that we will get a reward, although conscious reflection tells us that the probability is that we will lose any ‘sure bet’. This is a fascinating paradox, and one that has mental health implications for some individuals, who are compelled to continue betting even though it is at the cost of their quality of life. As psychiatrists, we are all aware that gambling can be an addiction, and neuroscientific research has shown that the neural processes involved are shared with other addictions, such as those associated with substance misuse.

Owing to the potential harms gambling can cause, at least to some individuals, most societies attempt to regulate it. Some religions explicitly prohibit gambling (Islam), or at the very least express grave concerns about it (Hinduism, Buddhism, Judaism, Christianity). As societies become more secular, our reservations about gambling become more focused on the age at which the behaviour begins, with a view to protecting children.

We have two articles about problem gambling in diverse cultures in this issue of *BJPsych International*, one focused on Nigeria^1^ and the other on Malaysia.^2^ The interface between governmental regulation and the urge to gamble is a key issue in both articles.

A total ban on gambling seems unrealistic in any society, as it will just be driven underground. But, if gambling is permitted, how does one ensure that it does not cause harm? Should governments encourage some forms of gambling, such as lotteries, as a way of raising money for good causes? Does permitted advertising of privately organised gambling activities (casinos, horse racing, sporting fixtures) reflect an open society? Or does the encouragement of gambling of any sort lead to abuse and, for some individuals, the road to penury? Both Nigeria and Malaysia are struggling with these challenging questions, as are we in the UK.
